# Temporal Trends in the Outcomes of Percutaneous Coronary Intervention With Zotarolimus Eluting Stents Versus Everolimus Eluting Stents: A Meta‐Analysis of Randomized Controlled Trials

**DOI:** 10.1002/clc.24306

**Published:** 2024-06-18

**Authors:** Jawad Basit, Mushood Ahmed, Aimen Shafiq, Zaofashan Zaheer, Abdulqadir J. Nashwan, Aleena Ahmed, Mohammad Hamza, Usman Naseer, Shafaqat Ali, Neelesh Gupta, Yasar Sattar, Akram Kawsara, Ramesh Daggubati, M. Chadi Alraies

**Affiliations:** ^1^ Department of Medicine Rawalpindi Medical University Rawalpindi Pakistan; ^2^ Cardiovascular Analytics Group Canterbury UK; ^3^ Department of Medicine Dow University of Health Sciences Karachi Pakistan; ^4^ Department of Medicine King Edward Medical University Lahore Pakistan; ^5^ Hamad Medical Corporation Doha Qatar; ^6^ Guthrie Medical Group Cortland New York USA; ^7^ Department of Cardiology USD Sanford School of Medicine Sioux Falls South Dakota USA; ^8^ Department of Internal Medicine Louisiana State University Shreveport Louisiana USA; ^9^ Department of Cardiology, Kirk Kerkorian School of Medicine University of Nevada Las Vegas Las Vegas Nevada USA; ^10^ Department of Interventional Cardiology West Virginia University Morgantown West Virginia USA; ^11^ Department of Cardiology, Detroit Medical Center Wayne State University Detroit Michigan USA

**Keywords:** Everolimus‐eluting stents (EES), major adverse cardiovascular events (MACE), meta‐analysis, randomized controlled trials, target vessel myocardial infarction (TVMI), Zotarolimus‐eluting stents (ZES)

## Abstract

**Introduction:**

Long‐term follow‐up results of various trials comparing Zotarolimus eluting stents (ZES) with Everolimus eluting stents (EES) have been published recently. Additionally, over the last decade, there have been new trials comparing the ZES with various commercially available EES. We aim to conduct an updated meta‐analysis in light of new evidence from randomized controlled trials (RCTs) to provide comprehensive evidence regarding the temporal trends in the clinical outcomes.

**Methods:**

A comprehensive literature search was conducted across PubMed, Cochrane, and Embase. RCTs comparing ZES with EES for short (<2 years), intermediate (2–3 years), and long‐term follow‐ups (3–5 years) were included. Relative risk was used to pool the dichotomous outcomes using the random effects model employing the inverse variance method. All statistical analysis was conducted using Revman 5.4.

**Results:**

A total of 18 studies reporting data at different follow‐ups for nine trials (*n* = 14319) were included. At short‐term follow‐up (<2 years), there were no significant differences between the two types of stents (all‐cause death, cardiac death, Major adverse cardiovascular events (MACE), target vessel myocardial infarction, definite or probable stent thrombosis or safety outcomes (target vessel revascularization, target lesion revascularization, target vessel failure, target lesion failure). At intermediate follow‐up (2–3 years), EES was superior to ZES for reducing target lesion revascularization (RR = 1.28, 95% CI = 1.05–1.58, *p* < 0.05). At long‐term follow‐up (3–5 years), there were no significant differences between the two groups for any of the pooled outcomes (*p* > 0.05).

**Conclusion:**

ZES and EES have similar safety and efficacy at short, intermediate, and long‐term follow‐ups.

## Graphical abstract

A meta‐analysis comparing the outcomes of percutaneous coronary intervention with Everolimus eluting stents versus Zotaralimus eluting stents. EES and ZES showed similar efficacy and safety at short‐, mid‐, and long‐term follow‐up. EES was found to be superior to ZES for target lesion revascularization at intermediate follow‐up.

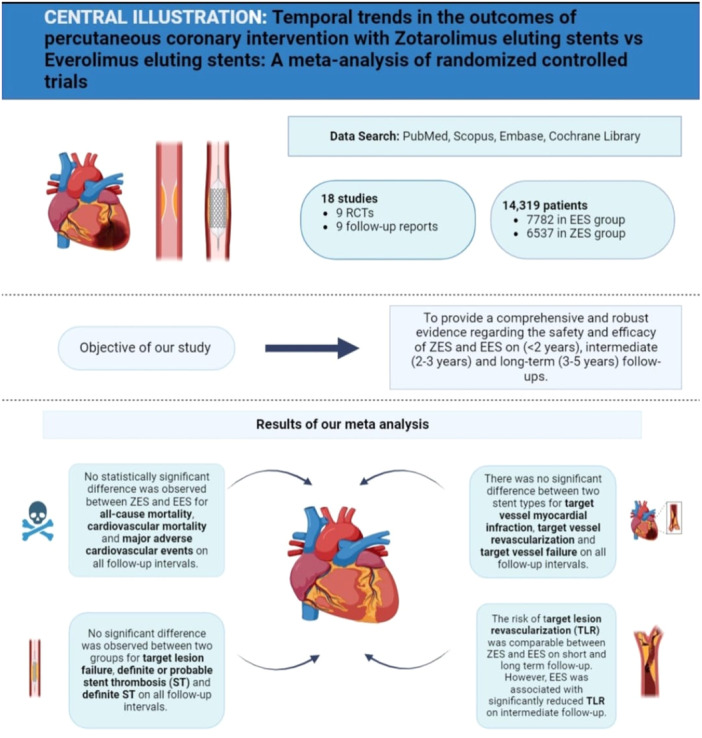

## 1 Introduction

Drug‐eluting stents (DES) have changed the paradigm of percutaneous coronary interventions ever since the harsh learnings from Bare Metal Stent (BMS) Era in the late 1990s [1, 2, 3]. Improvements in stent design have resulted in clinically improved outcomes albeit at the expense of stent thrombosis (ST) or very late ST as in the case of BMS and first‐generation DES [3, 4, 5]. However, the knowledge of the persistence of adverse events with both first‐generation and contemporary DES presents an opportunity for improvement [6]. Specifically, dedicated longitudinal follow‐up in clinical trials offers greater insight into the effectiveness of DES, and the accrual of events may amplify the ability to distinguish outcomes between therapies [7]. As an example, it was long‐term surveillance of first‐generation DES that introduced the risk associated with late ST, and detailed patient‐level observation for years beyond a trial's primary endpoint [4]. This has now become a regulatory mandate. Detailed ascertainment of events permits insight into not only annualized estimates of stent thrombosis but also the persistence of late target lesion revascularization that seems constant with existing DES.

Coronary interventions cause endothelial disruption and vascular injury that leads to the initiation of healing processes through mitogen‐mediated proliferation of vascular smooth muscle cells (VSMC) and migration of these smooth muscle cells from the media to the intima [8]. Currently, available anti‐restenotic drugs primarily target this which is the key event for the formation of neointimal hyperplasia and restenosis [8, 9]. First‐generation DES and second‐generation DES differ in the drug used to achieve this anti‐restenotic effect which can attributed to differences in drug solubility and mean tissue concentration [10, 11]. These drugs also differ in terms of structure, molecular weight, potency, and lipophilicity. Zotarolimus and Everolimus are sirolimus analogs that have a lipophilic tetrazole and hydroxyethyl group instead of the hydroxyl group at position 40 of the sirolimus, respectively [12, 13]. Zotarolimus is a highly lipophilic analog of sirolimus and was designed for vascular injury [14]. Everolimus has a much higher interaction with the mechanistic target of rapamycin complex 2, higher bioavailability, and shorter half‐life than sirolimus and has shown more rapid endothelization [15].

With regard to the Everolimus eluting stents (EES) group, there also exists a considerable range of differences in drug concentration, complete drug elution time, polymer coating (durable, bioabsorbable), stent platform, strut shape (round or square) links/connectors and finally, its composite thickness and that of the stent and the polymer [16, 17]. The BioLink polymer in R‐Zotarolimus eluting stents (ZES), which is made up of three polymers—a hydrophobic C10 polymer, a hydrophilic C19 polymer, and water‐soluble polyvinyl pyrrolidinone—and the two‐layer polymer system in EES, which has an acrylate primer and a fluorinated copolymer, both release antiproliferative agents from biocompatible durable polymers [9, 13].

Data regarding the safety and efficacy of both stent types is limited. Previous meta‐analyses did not assess clinical outcomes beyond the 2–3‐year follow‐up or were limited in the number of pooled studies [18, 19]. Only recently we have had access to 5‐year follow‐up data [7, 20]. There is no head‐to‐head comparison of Resolute—ZES (Medtronic) and the composite of all different commercially available stents with EES as well. Considering this literature gap, we conducted the present study that has systematically synthesized evidence regarding the safety and efficacy of ZES compared with EES on short‐, intermediate‐, and long‐term follow‐ups. Our meta‐analysis comprises all comers PCI using ZES/EES including acute coronary syndromes, left ventricular dysfunction, diabetes mellitus, and small vessels.

## 2 Methods

This systematic review and meta‐analysis was conducted following the guidelines established by Preferred Reporting Items for Systematic Review and Meta‐Analysis (PRISMA) [21] and the Cochrane Handbook of Systematic Reviews [22].

### 2.1 Data Sources and Search Strategy

Two authors (A.A. and M.A.) independently searched PubMed/MEDLINE, Scopus, Embase, the Cochrane Library, and ClinicalTrials.gov from their inception till October 1, 2023. No language restrictions were applied. The aim was to identify all randomized controlled trials (RCTs) and their follow‐up reports that aimed to assess the safety and efficacy of ZES compared with EES. The researchers screened references from retrieved trials, previous meta‐analyses, and review articles to identify all studies that fulfill the eligibility criteria. The detailed search strategy for three databases is provided in Supporting Information S1: Table 1.

The studies were eligible for inclusion in our systematic review and meta‐analysis if they were: (i) RCTs (ii) Short (<2 years), Intermediate (2–3 years), or long‐term (3–5 years) follow‐up reports of RCTs; (iii) RCTs that included adult male or female patients who were >18 years old; (iv) compared clinical outcomes of ZES with EES; (v) assessed at least one of the predetermined clinical outcomes. In the case of trials reporting outcomes at two different intervals for the same follow‐up strata (e.g., for long‐term follow‐up, if outcomes were separately reported for 4‐ and 5‐year follow‐up, the data was extracted and pooled for longer follow‐up). Post hoc analyses of parent RCTs were excluded. The detailed inclusion and exclusion criteria of our meta‐analysis are given in Supporting Information S1: Table 2.

The primary outcome was safety assessed in terms of all‐cause death, cardiac death, major adverse cardiovascular events (MACE), target vessel myocardial infarction (MI), definite or probable stent thrombosis (ST), and definite ST while the secondary outcome was efficacy gauged in terms of target vessel revascularization (TVR), target lesion revascularization (TLR), target vessel failure (TVF), and target lesion failure (TLF). The detailed definitions of included outcomes are given in Supporting Information S1: Table 3. All outcomes were assessed on short (<2 years), intermediate (2–3 years), and long‐term (3–5 years) follow‐ups.

### 2.2 Study Selection, Data Extraction, and Quality Assessment

The studies identified from the literature search were imported to EndNote X9 (Clarivate Analytics), and duplicate records were removed. Two researchers (A.A. and M.A.) independently reviewed the studies based on their titles and abstracts. This was followed by a review of the full‐text of the studies, and any study that fulfilled our predetermined eligibility criteria was included. A third author (J.B.) was consulted in case of any disagreement.

We extracted the following data from each study: author surname, publication year, number of patients in ZES and EES groups, mean age of patients, percentage of males among the study participants, details of patient population, intervention subtype, use of dual antiplatelet therapy, duration of follow‐up, and primary and secondary outcomes.

The Cochrane risk of bias tool (RoB 2.0) was used for assessing the risk of bias for the included RCTs [23]. The risk of bias was assessed across the following domains: randomization, deviations from intended variation, missing outcome data, measurement of outcome, and selection of reported results. The trials were scored based on a high, some concerns, or low risk of bias in each domain. Traffic light plots were created using the Robvis tool [24].

### 2.3 Statistical Analysis

RevMan Version 5.4 (Nordic Cochrane Center, Copenhagen, Denmark) was used for data analysis. We used risk ratio (RR) along with 95% confidence intervals (CIs) to present results. DerSimonian and Laird random effects model was used to pool results [25] and forest plots were generated to visualize the results. Higgins *I* ^2^ test was used to assess heterogeneity across the included studies; low heterogeneity indicated by a value of <25%; moderate heterogeneity indicated by 25%–75%; and >75% indicated high heterogeneity [26]. Begg's rank test [27] and Egger's regression test [28] were conducted to assess the risk of publication bias. A *p‐*value of <0.05 was considered significant in all cases.

## 3 Results

**Figure 1:**
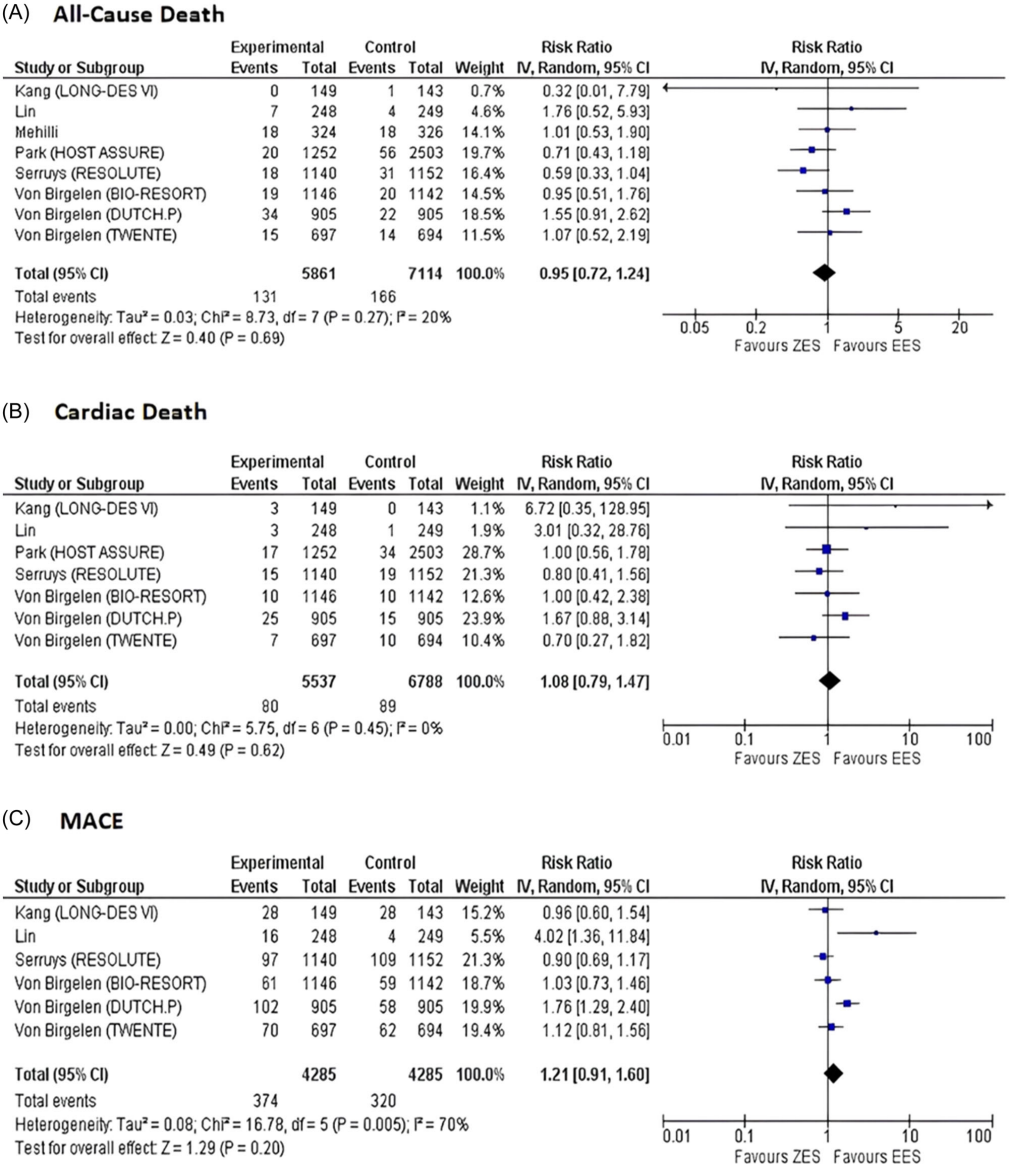
Forest plots for pooled (A) all‐cause death, (B) cardiac death, and (C) MACE on Short‐term follow‐up. MACE, major adverse cardiovascular events.

We conducted a systematic literature search using PubMed/Medline, Embase, and Cochrane Library that yielded X records. Duplicate studies were removed, and screening of articles was performed based on title, abstract, and full text. After screening, 18 studies were found eligible to be included in our meta‐analysis. The study selection process is shown in Supporting Information S1: Figure 1. The detailed reasons for the exclusion of different RCTs during full‐length screening are given in Supporting Information S1: Table 4.

A total of 18 [7, 20, 29, 30, 31, 32, 33, 34, 35, 36, 37, 38, 39, 40, 41, 42, 43, 44] reports presenting data from 9 RCTs [29, 30, 31, 32, 33, 34, 35, 36, 37] for different follow‐ups were included. The details of included trials and their follow‐up reports are given in Supporting Information S1: Table 5. Our study included 7782 patients in the ZES group and 6537 in the EES group. The included studies were published from 2010 to 2022. The mean age of the participants ranged from 63.5 to 70.2 years. Male patients were more prevalent in all studies as they comprised >65% of the included participants in each study. Patients also received dual antiplatelet therapy that mainly consisted of clopidogrel with or without aspirin for 12 months in most studies. The follow‐up interval was divided into short (<2 years), intermediate (2–3 years) and long‐term periods (3–5 years). The detailed baseline characteristics of the included studies and participants are given in Table 1.

**Table 1:** Baseline characteristics of included studies and participants.

Author	Year	Number of patients, ZES/EES, *n*	Mean age, ZES/EES, mean (SD or IQR)	Male, ZES/EES, %	Patient population	Intervention subtype	DAPT use	Follow‐up
Ploumen et al.	2022	1173/1172	63.6 (10.9)/64.0 (10.7)	72.3/72.1	Patients presenting with any coronary syndrome could participate, and any type of lesion (e.g., de novo, restenotic, or coronary bypass lesion) was permitted	Synergy EES Resolute Integrity ZES	ZES = 43EES = 37	5 years
Youn et al.	2020	639/638	64.0 ± 11.3/65.6 ± 11.0	67.1/66.1	Patients with stable coronary artery disease or ACS including ST‐segment–elevation MI were eligible if at least 1 coronary artery had significant stenosis.	Xience EES Resolute ZES	ZES = 596EES = 590	2 years
Buiten et al.	2019	1173/1172	63·6 ± 10·9/64·0 ± 10·7	72.3/72.1	All coronary syndromes, de novo andrestenotic lesions, and coronary or bypass lesions were permitted	Very‐thin strut platinum‐chromium Synergy EUSdurable‐polymer thin‐strut cobalt chromium Resolute Integrity ZES	NR	3 years
Kim et al.	2019	1252/2503	60.78 ± 9.68/63.1 ± 10.8	65.6/69.8	Major exclusion criteria were severe left ventricular systolic dysfunction, cardiogenic shock, symptomatic heart failure, and an increased risk of bleeding	Platinum Chromium EUS Cobalt Chromium ZES	ZES = 37 EES = 55	3 years
Kang et al.	2019	153/149	65.8 ± 8.6/65.9 ± 10.0	75.2/73.2	Patients with very long coronary artery disease of diameter stenosis of at least 50% and visual lesion length of atleast 50 mm who planned to implant at least two DES were enrolled in the study. Exclusion criteria included acute ST‐segment MI necessitating primary PCI	Resolute R‐ZES Xience EES	All patients on DAPT for at least 12 months	13 months
Zocca et al.	2018	906/905	63.9 ± 10.6/63.9 ± 11.0	73.4/72.6	Patients with an indication for PCI (all coronary syndromes, de novo and restenotic lesions, and coronary artery or bypass stenosis were permitted)	ZES (cobalt chromium‐based Resolute Integrity) EES (platinum chromium‐based PROMUS Element)	ZES = 262 EES = 259	5 years
Kok et al.	2018	1173/1172	63.6 ± 10.9/64.0 ± 10.7	72.3/72.1	All coronary syndromes, de novo and restenotic lesions, and coronary or bypass lesions were permitted. There was no limit for lesion length, reference vessel size, and number of lesions or vessels to be treated	Platinum‐chromium EES (SYNERGY), new generation thin‐strut ZES (Resolute Integrity)	ZES = 103 EES = 87	2 years
von Birgelen et al.	2016	1173/1172	63.6 ± 10.9/64.0 ± 10.7	72.3/72.1	Patients presenting with any coronary syndrome could participate, and any type of lesion (e.g., de novo, restenotic, or coronary bypass lesion) was permitted	Platinum‐chromium EES (SYNERGY), new generation thin‐strut ZES (Resolute Integrity)	All patients on DAPT for 6‐12 months	1 year
Sen et al.	2015	906/905	63.9 ± 10.6/63.9 ± 11.0	73.4/72.6	Patients aged 18 years and older who required a PCI with implantation of a DES were recruited	Resolute Integrity ZES Promus Element EES	ZES = 262 EES = 259	2 years
Lin et al.	2015	248/249	62 (55–71)/65 (57–72)	73.79/74.3	Patients were eligible if they had at least one coronary lesion with stenosis of more than 50% in a vessel with a reference diameter of 2.25 to 4.0 mm.	ZES, Abbott EES, Medtronic	Clopidogrel for 12 months	15 months
Taniwaki et al.	2014	1122/1124	64.4 ± 10.9/64.2 ± 10.8	76.7/77.2	Patients with stable CAD or acute coronary syndromes requiring revascularization	Resolute‐ZES (Medtronic, Minneapolis, Minnesota) and XIENCE V EES (Abbott Vascular, Abbott Park, Illinois)	ZES = 124 EES = 121	4 years
Park et al.	2014	2,503/1,252	63.5 ± 10.7/63.1 ± 10.8	65.6/69.8	Trial participants were 18 years of age or older and had at least one clinically significant stenotic lesion amenable to PCI in the coronary artery, venous or arterial bypass grafts	platinum‐chromium EES; cobalt‐chromium ZES	Patients were randomized to TAT or DDAT	1 year
Lowik et al.	2014	692/689	63.9 ± 10.9/64.5 ± 1 10.7	72.5/72.6	Patients with stable angina or non‐ST‐elevation acute coronary syndromes	Second‐Generation Zotarolimus‐Eluting Resolute and Everolimus‐Eluting Xience V Stents	NR	3 years
Tandjung et al.	2013	697/694	63.9 ± 10.9/64.5 ± 10.7	72.5/72.6	Indication for percutaneous coronary intervention (PCI) with DES	Second‐Generation Zotarolimus‐Eluting Resolute and Everolimus‐Eluting Xience V Stents	All patients on 12 months DAPT	2 years
von Birgelen et al.	2013	905/905	64 (56–72)/65 (57–72)	73/73	Patients aged 18 years and older who required a PCI with implantation of a DES were recruited	Resolute Integrity ZES Promus Element EES	ZES = 262 EES = 259	1 year
Mehilli et al.	2013	324/326	69.4 ± 10.4/70.2 ± 9.4	72.8/77.3	Patients older than 18 years of age with ischemic symptoms or evidence of myocardial ischemia in the presence of 50% de novo stenosis located in the left main stem	Resolute ZES (Medtronic Cardiovascular, Santa Rosa, California) Xience V EES (Abbott Vascu‐lar Devices, Santa Clara, California)	clopidogrel 75 mg/day or prasugrel 10 mg/day for at least 12 months Aspirin indefinitely	1 year
von Birgelen et al.	2012	697/694	63.9 ± 10.9/64.5 ± 10.7	72.5/72.6	Acute ST‐segment elevation myocardial infarctions not eligible	Resolute ZES (Medtronic Cardiovascular, Santa Rosa, California) Xience V EES (Abbott Vascular Devices, Santa Clara, California)	NR	
Serruys et al.	2010	1140/1152	64.4 ± 10.9/64.2 ± 10.8	76.7/77.2	Patients with chronic, stable coronary artery disease or acute coronary syndromes, including myocardial infarction with or without ST‐segment elevation	Resolute ZES (Medtronic CardioVascular) EES (Xience V, Abbott Vascular Devices)	All patients on 75 mg of acetylsalicylic acid indefinitely 75 mg of clopidogrel for 6 months	13 months

*Note:* Number of patients who received ZES = 7782. Number of patients who received EES = 6537. Weighted average of mean age ZES/EES = 64.1 ± 9.9/65 ± 10.0. Weighted average (%) of male population ZES/EES = 72.1/73.4.

Abbreviations: CAD, coronary artery disease; DES, drug eluting stents; EES, Everolimus‐eluting stent; IQR, Interquartile range; *n*, number; NR, not reported; PCI, percutaneous coronary intervention; SD, standard deviation; ZES, Zotarolimus‐eluting stent.

### 3.1 Quality Assessment and Publication Bias in Included Trials

**Figure 2:**
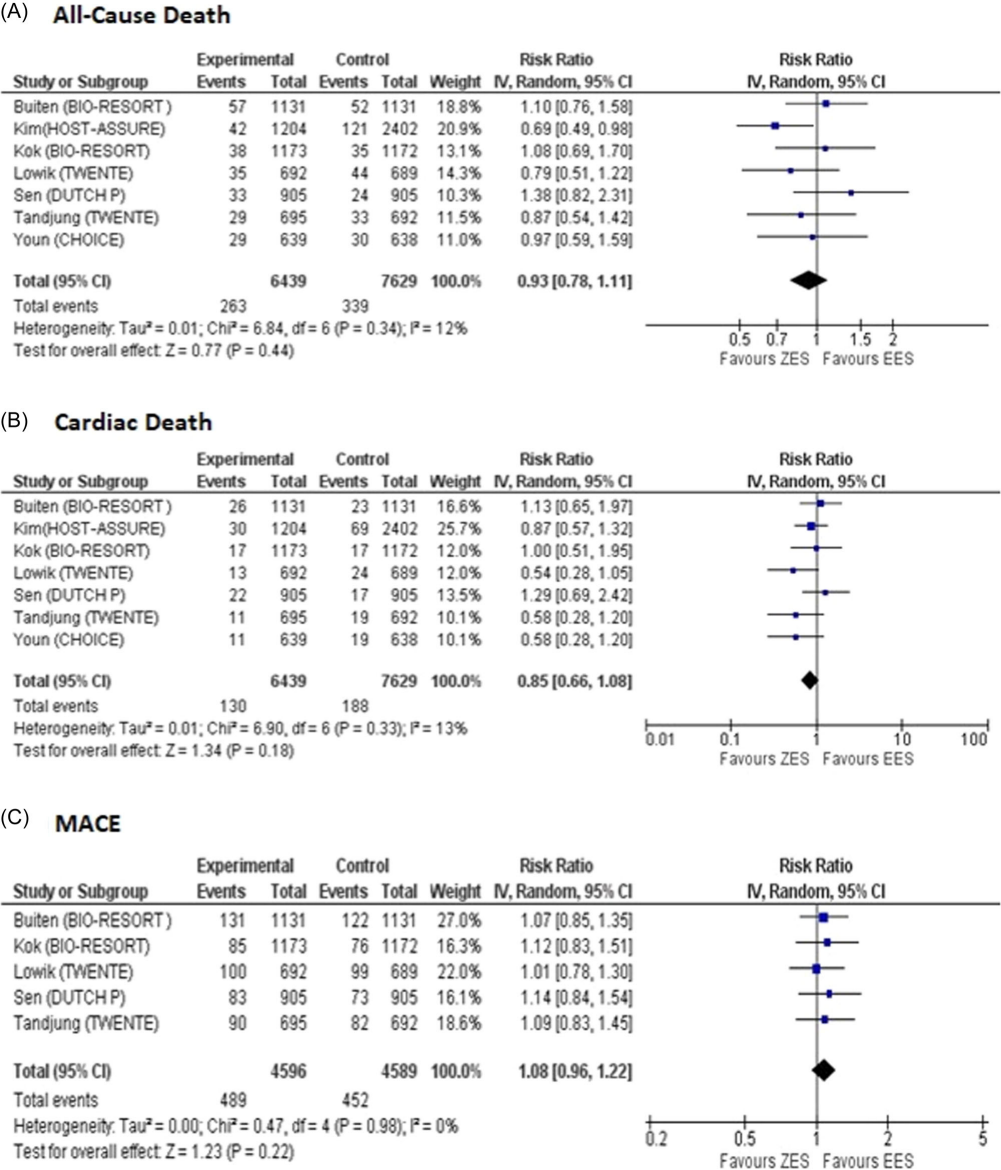
Forest plots for pooled (A) all‐cause death, (B) cardiac death, and (C) MACE on Intermediate follow‐up. MACE, major adverse cardiovascular events.

**Figure 3:**
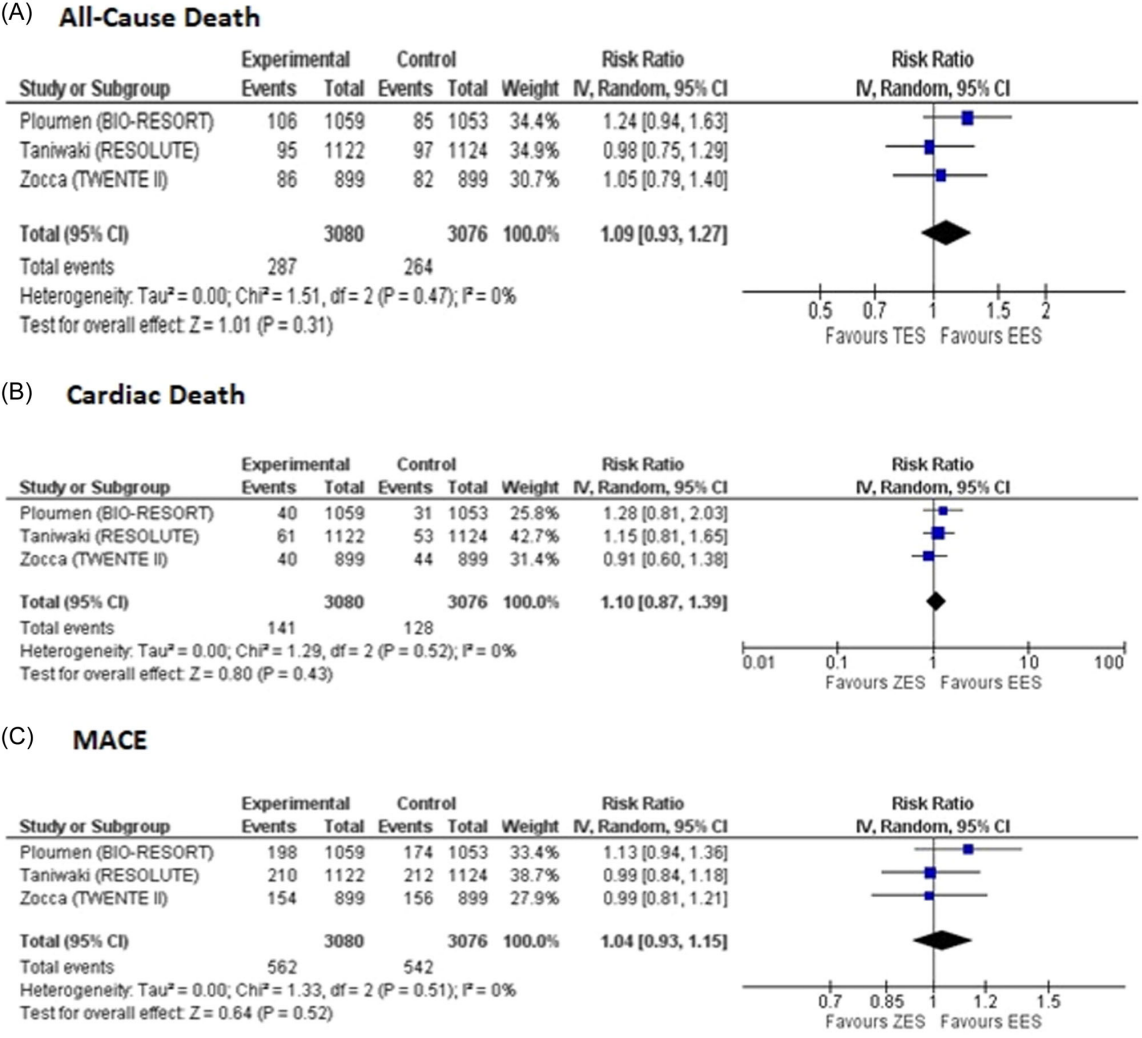
Forest plots for pooled (A) all‐cause death, (B) cardiac death, and (C) MACE on Long‐term follow‐up. MACE, Major adverse cardiovascular events.

Most of the RCTs were found to be at low risk of bias while two had high risk of bias. The details of the bias assessment for each trial are given in Supporting Information S1: Figure 2. The publication bias was assessed for 30 pooled outcomes. None of the pooled outcomes showed significant publication bias except TLR at long‐term follow‐up. The detailed results the Begg's rank and Egger's regression test for each outcome are given in Supporting Information S1: Tables 6–8. The funnel plots for all‐cause death, cardiac death, and MACE are given in Supporting Information S1: Figure 3 **.**

### 3.2 Results of Meta‐Analysis

Clinical outcomes were assessed for short (<2 years), intermediate (2–3 years), and long‐term (3–5 years) follow‐ups.

#### 3.2.1 Clinical Outcomes on Short‐Term Follow‐Up (<2 years)

##### 3.2.1.1 All‐Cause Death

Data for all‐cause death was provided by eight studies for the ZES group (events, 131; total, 5861) and the EES group (events, 166; total, 7114). Our pooled analysis showed that there was no significant difference between both groups for reduced risk of all‐cause death on short‐term follow‐up (RR = 0.95, 95% CI: 0.72 to 1.24, *p* = 0.69 Figure 1A). Low heterogeneity was observed among the included studies for this outcome (*I* ^2^ = 20%).

##### 3.2.1.2 Cardiac Death

Our pooled analysis of seven studies providing data for cardiac deaths in the ZES group (events, 80; total, 5537) and EES group (events, 89; total, 6788) demonstrated that the risk of cardiac death was comparable in both groups with no heterogeneity observed across the included studies (RR = 1.08, 95% CI: 0.79 to 1.47, *p* = 0.62, *I* ^2^ = 0% Figure 1B).

##### 3.2.1.3 MACE

Out of 18 eligible studies, data for MACE on short‐term follow‐up was provided by six studies for ZES group (events, 374; total, 4285) and EES group (events, 320; total, 4285). No significant difference was observed between ZES and EES for MACE (RR = 1.21, 95% CI: 0.91 to 1.60, *p* = 0.20 Figure 1C). However, we observed a moderate risk of heterogeneity for this outcome (*I* ^2^ = 70%).

##### 3.2.1.4 Target Vessel Myocardial Infraction (MI)

**Figure 4:**
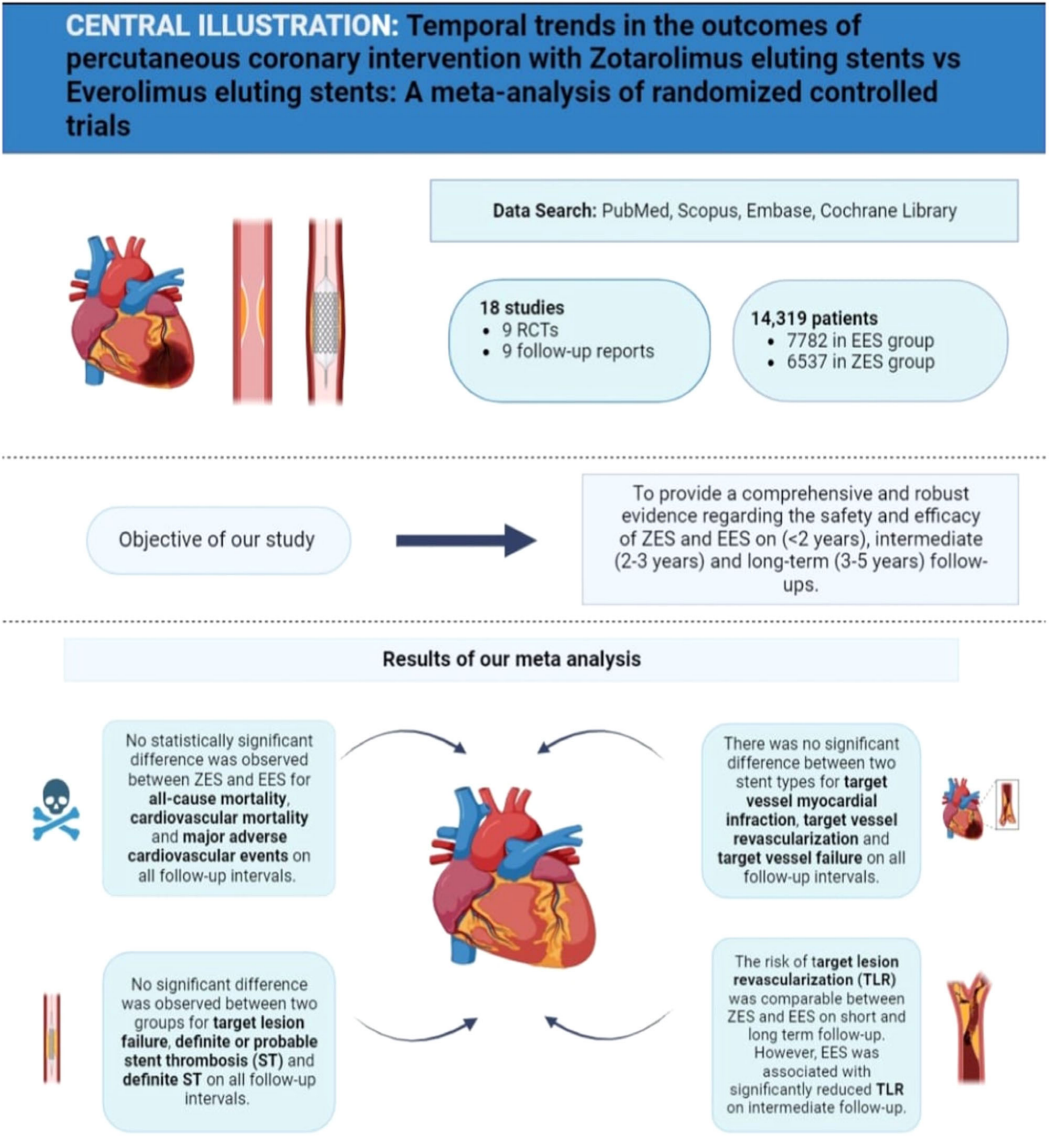
Central illustration summarizing the findings of our meta‐analysis.

Target‐vessel MI was reported by 6 out of 18 studies for the ZES group (events, 177; total, 5289) and EES group (events, 173; total, 6539). There was no significant difference between both groups for target vessel MI with no observed heterogeneity (RR = 1.09, 95% CI: 0.89 to 1.34, *p* = 0.42, *I* ^2^ = 0%, Supporting Information S1: Figure 4A).

###### 3.2.1.4.1 Definite or Probable ST

We pooled data reported by six studies for definite or probable ST in ZES group (events, 44; total, 4285) and EES group (events, 28; total, 4285) that showed no significant difference between ZES and EES (RR = 1.53, 95% CI: 0.89 to 2.64, *p* = 0.13, *I* ^2^ = 14%, Supporting Information S1: Figure 4B).

###### 3.2.1.4.2 Definite ST

Only two studies reported incidence of short‐term definite ST in the ZES group (events, 12; total, 2051) and the EES group (events, 7; total, 2047). On pooling the available data, the risk of definite ST was comparable across both groups with a moderate risk of heterogeneity (RR = 1.57, 95% CI: 0.40 to 6.12, *p* = 0.51, *I* ^2^ = 47%, Supporting Information S1: Figure 4C).

##### 3.2.1.5 TVR

On pooling six studies that provided data for the ZES group (events, 209; total, 5289) and the EES group (events, 190; total, 6539) no significant difference was detected between both groups for the occurrence of TVR (RR = 1.22, 95% CI: 0.97 to 1.53, *p* = 0.09, *I* ^2^ = 27%, Supporting Information S1: Figure 5A).

##### 3.2.1.6 TLR

The incidence of TLR on short‐term follow‐up was provided by eight studies for the ZES group (events, 211; total, 5861) and the EES group (events, 174; total, 7114). On pooling the reported data, no significant difference was found between both groups for this outcome. Heterogeneity was moderate for short‐term TLR across the pooled studies (RR = 1.29, 95% CI: 0.99 to 1.68, *p* = 0.06, *I* ^2^ = 38%, Supporting Information S1: Figure 5B).

##### 3.2.1.7 TVF

Short‐term TVF was reported by 5 studies for the ZES group (events, 318; total, 5140) and the EES group (events, 348; total, 6396). ZES had no significant difference as compared with EES for reduction in TVF (RR = 1.04, 95% CI: 0.89 to 1.20, *p* = 0.64, *I* ^2^ = 0%, Supporting Information S1: Figure 5C).

##### 3.2.1.8 TLF

TLF was reported in six studies for short‐term follow‐up in the ZES group (events, 356; total, 5289) and the EES group (events, 341; total, 6539). No significant difference was observed between both groups for short‐term TLF (RR = 1.15, 95% CI: 0.94 to 1.41, *p* = 0.18, *I* ^2^ = 47%, Supporting Information S1: Figure 5D).

#### 3.2.2 Clinical Outcomes on Intermediate Follow‐Up Period (2–3 years)

##### 3.2.2.1 All‐Cause Death

Our pooled analysis of seven studies that provided data for all‐cause death on intermediate follow‐up showed that there was no significant difference between both groups for reduced risk of all‐cause death (RR = 0.93, 95% CI: 0.78 to 1.11, *p* = 0.44, *I* ^2^ = 12% Figure 2A).

##### 3.2.2.2 Cardiac Death

Out of 18 eligible studies, seven reported cardiac death in the ZES group (events, 130; total, 6439) and the EES group (events, 188; total, 7629). ZES had no significant difference compared to EES for reduced risk of cardiac death (RR = 0.85, 95% CI: 0.66 to 1.08, *p* = 0.18, *I* ^2^ = 13% Figure 2B).

##### 3.2.2.3 MACE

Data regarding MACE was reported by five studies for the ZES group (events, 489; total, 4596) and the EES group (events, 452; total, 4589). There was no significant difference between both groups for this outcome and no heterogeneity was observed (RR = 1.08, 95% CI: 0.96 to 1.22, *p* = 0.22, *I* ^2^ = 0%, Figure 2C).

##### 3.2.2.4 Target Vessel MI

A pooled analysis of seven studies that reported intermediate target vessel MI for the ZES group (events, 193; total, 6439) and EES group (events, 185; total, 7629) showed no significant difference between both groups (RR = 1.08, 95% CI: 0.88 to 1.32, *p* = 0.46, *I* ^2^ = 0%, Supporting Information S1: Figure 6A).

##### 3.2.2.5 Definite or Probable ST

Intermediate definite or probable ST was reported by seven studies for the ZES group (events, 59; total, 6439) and the EES group (events, 70; total, 7629). No significant difference was observed between both groups for this outcome (RR = 0.93, 95% CI: 0.66 to 1.32, *p* = 0.69, *I* ^2^ = 0%, Supporting Information S1: Figure 6B).

##### 3.2.2.6 Definite ST

Out of 18 selected studies, 6 reported definite ST for the ZES group (events, 36; total, 5235) and EES group (events, 27; total, 5227). Our pooled analysis demonstrated no significant difference between both groups for definite ST on intermediate follow‐up (RR = 1.32, 95% CI: 0.70 to 2.48, *p* = 0.40, *I* ^2^ = 25%, Supporting Information S1: Figure 6C).

##### 3.2.2.7 TVR

Data for TVR on intermediate follow‐up was provided by six studies. There was no significant difference between ZES and EES for the occurrence of TVR (RR = 1.20, 95% CI: 0.98 to 1.46, *p* = 0.08, *I* ^2^ = 24%, Supporting Information S1: Figure 7A).

##### 3.2.2.8 TLR

On pooling data reported by seven studies for ZES group (events, 249; total, 6487) and EES group (events, 240; total, 7730) for intermediate TVR that there was a significantly reduced risk for TLR in EES group as compared to ZES group (RR = 1.28, 95% CI: 1.05 to 1.56, *p* = 0.02, Supporting Information S1: Figure 7B). Heterogeneity remained low across the pooled studies (*I* ^2^ = 16%).

##### 3.2.2.9 TVF

Out of 18 eligible studies, 5 reported data for TVF on intermediate follow‐up showed that there was no significant difference between both groups for a reduction in TVF (RR = 1.05, 95% CI: 0.93 to 1.20, *p* = 0.41, *I* ^2^ = 0%, Supporting Information S1: Figure 7C).

##### 3.2.2.10 TLF

Data regarding TLF was provided by six studies for the ZES group (events, 467; total, 5800) and the EES group (events, 493; total, 6991). The risk of TLF remained comparable between both groups (RR = 1.09, 95% CI: 0.96 to 1.23, *p* = 0.18, *I* ^2^ = 0%,Supporting Information S1: Figure 7D).

#### 3.2.3 Clinical Outcomes on Long‐Term Follow‐Up (3–5 years)

##### 3.2.3.1 All‐Cause Death

Long‐term all‐cause death was reported by three studies for the ZES group (events, 287; total, 3080) and EES group (events, 264; total, 3076). The incidence of all‐cause death remained comparable with a moderate risk of heterogeneity (RR = 1.09, 95% CI: 0.93 to 1.27, *p* = 0.31; *I* ^2^ = 0% Figure 3A).

##### 3.2.3.2 Cardiac Death

On pooling three studies that reported data for long‐term cardiac deaths for the ZES group (events, 141; total, 3080) and the EES group (events, 128; total, 3076) no significant difference was observed (RR = 1.10, 95% CI: 0.87 to 1.39, *p* = 0.43, *I* ^2^ = 0% Figure 3B).

##### 3.2.3.3 MACE

Out of 18 included studies, 3 studies reported data for long‐term MACE for the ZES group (events, 562; total, 3080) and the EES group (events, 542; total, 3076). Our pooled analysis demonstrated that there was no significant difference between both groups for reducing MACE (RR = 1.04, 95% CI: 0.93 to 1.15, *p* = 0.52, *I* ^2^ = 0% Figure 3C).

##### 3.2.3.4 Target Vessel MI

A pooled analysis of three studies that reported long‐term target‐vessel MI for the ZES group (events, 138; total, 3080) and the EES group (events, 128; total, 3076) showed that there was no significant difference between both groups (RR = 1.08, 95% CI: 0.85 to 1.36, *p* = 0.54, *I* ^2^ = 0%, Supporting Information S1: Figure 8A).

##### 3.2.3.5 Definite or Probable ST

Our pooled analysis of three studies that reported data for long‐term definite or probable ST for the ZES group (events, 58; total, 3080) and the EES group (events, 45; total, 3076) showed no significant difference between ZES and EES (RR = 1.29, 95% CI: 0.87 to 1.89, *p* = 0.20, Supporting Information S1: Figure 8B). There was no heterogeneity in the results (*I* ^2^ = 0%).

##### 3.2.3.6 Definite ST

Long‐term definite ST was reported by three studies for the ZES group (events, 40; total, 3080) and the EES group (events, 29; total, 3076). No significant difference was observed between both groups along with no observed heterogeneity (RR = 1.36, 95% CI: 0.84 to 2.20, *p* = 0.21, *I* ^2^ = 0%, Supporting Information S1: Figure 8C).

##### 3.2.3.7 TVR

Data for TVR was provided by three studies for the ZES group (events, 316; total, 3080) and the EES group (events, 288; total, 3076). Our pooled analysis showed that there was no significant difference between both groups for the occurrence of TVR (RR = 1.09, 95% CI: 0.91 to 1.32, *p* = 0.36, *I* ^2^ = 32%, Supporting Information S1: Figure 9A).

##### 3.2.3.8 TLR

Long‐term TLR was reported by two studies for the ZES group (events, 165; total, 2181) and the EES group (events, 140; total, 2177). There was no significant difference between both groups (RR = 1.18, 95% CI: 0.95 to 1.46, *p* = 0.14, *I* ^2^ = 0%, Supporting Information S1: Figure 9B).

##### 3.2.3.9 TVF

Out of 18 eligible studies, 3 provided data for TVF on long‐term follow‐up for the ZES group (events, 473; total, 3080) and EES group (events, 449; total, 3076). No significant difference was observed between both groups for a reduction in TVF (RR = 1.05, 95% CI: 0.92 to 1.20, *p* = 0.47, *I* ^2^ = 22%, Supporting Information S1: Figure 9C).

##### 3.2.3.10 TLF

Data regarding TLF was provided by 3 studies for the ZES group (events, 408; total, 3080) and EES group (events, 386; total, 3076). Our pooled analysis showed that there was no significant difference between both groups for this outcome (RR = 1.06, 95% CI: 0.93 to 1.20, *p* = 0.42, *I* ^2^ = 0%, Supporting Information S1: Figure 9D). AMSTAR 2.0 checklist was used to guide the reporting of this review (Supporting Information S1: Figure 10). The results of meta‐analysis are summarized in central illustration Figure 4.

## 4 Discussion

Our meta‐analysis is a direct head‐to‐head comparison between ZES and EES with outcomes reported over varying follow‐up periods. A total of 18 reports consisting of data from 9 trials and over 3 follow‐ups were included. The pooled analysis showed that throughout the three distinct follow‐up periods—short (<2 years), intermediate (3–5 years), and long (>5 years), ZES and EES had a similar safety profile with comparable results for all‐cause death, cardiac death, MACE, target vessel MI, and stent thrombosis. The efficacy of the two DES was also comparable with no significant differences for TVR, TVF, and TLF. However, on intermediate follow‐up, there was a significantly reduced risk of TLR in the EES group but no significant differences were observed in TLR at short and long follow‐up.

Similar findings were demonstrated in a previously published meta‐analysis that pooled five studies [18] by Piccolo et al. in 2015. This study found no significant difference in relative risk when powered for TVR and ST between the two groups. Additionally, another meta‐analysis [19] by Gu et al. in 2015 separately pooled the data from RCTs as well as observational studies. The results of that study revealed that in RCTs, EES and ZES showed comparable safety (MACE, all‐cause mortality, nonfatal MI, ST) and efficacy (TVR, TVF, TLF), while in observational studies, EES was safer and more efficacious than ZES in terms of MACE, ST, TVR, TLR, and TLF. Whereas the pooled RCT and observational studies results indicated that EES was safer and more effective than ZES, with a lower risk of MACE, stent thrombosis, TVR, TLR, and TLF.

Consequently, these conflicting findings and limited clinical follow‐up warranted the need for a comprehensive analysis based on larger clinical trials with various follow‐ups to increase the power of the pooled analysis. Our efforts in this meta‐analysis purport the above evidence by demonstrating no difference in all causes of death, MACE, TLR, TVF, TVMI, definite/probable ST, and definite ST in short, intermediate, and long‐term follow‐up except for less TLR with EES as compared to ZES at intermediate follow up. We achieved this by including all comers receiving PCI with ZES/EES. TLR was noted to favor EES over ZES at intermediate‐term follow‐up but not at long‐term follow‐up. However, TLF, definite or probable ST, ST, or TVF did not differ at any follow‐up interval. It is difficult to ascertain the reasons for such a difference in effect shown at the intermediate follow‐up period.

More than 80% of PCI patients receive DES, which is the accepted standard of treatment in modern clinical practice [45]. Recent advancements in stent design, comparable or greater anti‐restenotic effectiveness, and steadily declining incidence of late ST have led to the replacement of old‐generation DES with new‐generation DES especially after 1 year [46, 47]. Therefore, other considerations, such as cost‐effectiveness, may play us guide preference.

One study [48] concluded that EES seems to be a cost‐effective treatment for patients with ST‐segment elevated myocardial infarction because of its incremental efficacy, even if its overall costs were greater than those of BMS. The estimated incremental cost‐effectiveness ratios were below the generally acceptable threshold values. Whereas another study [49] demonstrated that when it came to cost‐reduction, second‐generation DES (Zotarolimus and Everolimus) was superior to BMS (saving €184 with the base case). This was mostly because there were fewer second revascularizations, no myocardial infarctions, and no stent thrombosis. However, since ZES and EES's cost efficiency has not been directly compared before, it is the area that should be the focus of future research. The studies comparing the efficacy of drug‐eluting stents have shown contrasting results. A meta‐showed superiority of everolimus‐eluting stents as they significantly reduced the risk of repeat revascularisation and definite stent thrombosis compared to sirolimus‐eluting stents [50]. However, the results were mainly based on short follow‐up parent RCTs without pooling the long‐term follow‐up data. Another study [18] showed comparable clinical outcomes with everolimus‐eluting stents and zotarolimus‐eluting stents. However, the investigators pooled only five RCTs and reported data at short‐term follow‐up. In contrast to the previously available reviews, we pooled 18 studies reporting data for 9 parent RCTs and comprehensively assessed clinical outcomes at 3 different follow‐up intervals short (<2 years), intermediate (2–3 years), and long‐term periods (3–5 years). This makes our meta‐analysis the first review to show comparable efficacy of everolimus‐eluting stents and zotarolimus‐eluting stents on three different follow‐up intervals along with pooling data from studies that had not been evaluated in previous analyses. We analyzed only these stent types as several studies had reported their data at different follow‐ups. However, it had not been assessed statistically in a direct head‐to‐head comparison across three follow‐up intervals.

Although meta‐analyses have been conducted in the past on this topic, our meta‐analysis offers a more holistic and updated view regarding the safety and efficacy of ZES compared with EES. We have comprehensively synthesized evidence by pooling recently published studies that had never been pooled in earlier meta‐analyses. Robust and meticulous data extraction from multiple studies across variable follow‐up intervals has enhanced our meta‐analysis statistical power, offering a better understanding of the temporal dynamics of ZES and EES. These temporal trends help evaluate the safety of stents across different follow‐ups. Our meta‐analysis has accurately assessed the current state of the literature regarding ZES versus EES on short‐, intermediate‐, and long‐term follow‐ups. Long‐term surveillance of ZES and EES in our study can help clinicians in future decision‐making regarding stent usage during PCI.

This study also has some limitations. First, this is a study‐level meta‐analysis, and the absence of patient‐level information made it challenging to evaluate potential effect modifiers. Another potential limitation of this research might be the inclusion of patients who had non‐resorbable polymer EES implants, which are older than the present‐day EES that employ resorbable polymer and are currently in use. It is important to mention that the differences in lesion complexity and duration of DAPT can also influence results. The post hoc analysis of RCTs will help us better understand the clinical outcomes in patients with complex lesions. Moreover, we observed risk of publication bias (Egger's test & Begg's test) for TLR at intermediate follow‐up.

## Author Contributions

**Jawad Basit:** conceptualization, methodology, visualization, supervision, data curation, formal analysis, writing original draft, reviewing, and editing. **Mushood Ahmed:** methodology, visualization, supervision, data curation, formal analysis, writing original draft, reviewing, and editing. **Aimen Shafiq:** data curation, writing and reviewing, software, visualization. **Zaofashan Zaheer:** data curation, formal analysis, visualization, software, review and editing. **Abdulqadir J. Nashwan:** supervision, visualization, validation, review and editing. **Mohammad Hamza:** software, visualization, writing the original draft. **Aleena Ahmed:** writing and reviewing, data curation. **Usman Naseer:** data curation, formal analysis, writing, and reviewing. **Shafaqat Ali:** review and editing, software, validation. **Neelesh Gupta:** Validation, writing original draft, review, and editing. **Yasar Sattar:** supervision, validation, review, and editing. **Akram Kawsara:** supervision, validation, review and editing. **M. Chadi Alraies:** supervision, validation, review, and editing. **Ramesh B. Daggubatti:** supervision, validation, review, and editing.

## Ethics Statement

The authors have nothing to report.

## Conflicts of Interest

The authors declare no conflicts of interest.

## Supporting information

Supporting information.

## Data Availability

The data that support the findings of this study are available from the corresponding author upon reasonable request.
