# Supplementary material for: Temporal Trends in the Outcomes of Percutaneous Coronary Intervention With Zotarolimus Eluting Stents Versus Everolimus Eluting Stents: A Meta‐Analysis of Randomized Controlled Trials
Source: Clin Cardiol. 2024 Jun 18;47(6):e24306. doi: 10.1002/clc.24306 (PMC11184471; doi:10.1002/clc.24306)
Supplement: Supplementary file 1 — Supporting information. [file CLC-47-e24306-s001.docx]

**Supplementary Appendix**

**Supplementary Figure 1:** PRISMA flow chart

**Supplementary Figure 2:** Risk of Bias in Included Trials using the ROB 2.0 tool

**Supplementary Figure 3:** Funnel plots for outcomes at short, intermediate, and long-term follow-up

**Supplementary Figure 4**: Forest plots showing pooled results at short-term follow-up (A) Target vessel Myocardial Infraction (B) Definite or probable stent thrombosis (ST) (C) Definite stent thrombosis (ST)

**Supplementary Figure 5**: Forest plots showing pooled results at short-term follow-up (A) Target vessel revascularization [TVR] (B) Target lesion revascularization [TLR] (C)Target vessel failure [TVF] (D) Target lesion failure [TLF]

**Supplementary Figure 6:** Forest plots showing pooled results at intermediate-term follow-up (A) Target vessel Myocardial Infraction [MI] (B) Definite or probable stent thrombosis [ST] (C) Definite stent thrombosis [ST]

**Supplementary Figure 7:** Forest plots showing pooled results at intermediate-term follow-up (A) Target vessel revascularization [TVR] (B) Target lesion revascularization [TLR] (C)Target vessel failure [TVF] (D) Target lesion failure [TLR]

**Supplementary Figure 8:** Forest plots showing pooled results at long-term follow-up (A) Target vessel Myocardial Infraction [MI] (B) Definite or probable stent thrombosis [ST] (C) Definite stent thrombosis [ST]

**Supplementary Figure 9:** Forest plots showing pooled results at intermediate-term follow-up (A) Target vessel revascularization [TVR] (B) Target lesion revascularization [TLR] (C)Target vessel failure [TVF] (D) Target lesion failure [TLR]

**Supplementary Figure 10:** AMSTAR 2.0 checklist

**Supplementary Table 1:** Search Strategies for online databases

**Supplementary Table 2:** Detailed inclusion and exclusion criteria

**Supplementary Table 3:** Definitions of pooled outcomes.

**Supplementary Table 4:** Reason for exclusion for studies during full length screening

**Supplementary Table 6:** Publication bias using Eggers and Begg’s test for short term follow-up

**Supplementary Table 7:** Publication bias using Eggers and Begg’s test for intermediate term follow-up

**Supplementary Table 8:** Publication bias using Eggers and Begg’s test for long term follow-up

**Supplementary Figure 1:** PRISMA flow chart

Duplicate records removed

(n = 843)

**Identifcation**

Records Identified through PubMed, Embase, and Cochrane Library

(Total = 2,003)

Records after duplicates

removed

(n = 1,160)

Records excluded after title and

abstract screening

(n = 941)

**Screening**

Full-text articles assessed for

eligibility

(n = 42)

Articles that did not meet inclusion criteria or had insufficient data

(n = 24)

**Included**

Studies included in

qualitative synthesis

(n = 18)

Studies included in quantitative synthesis

(n= 18)

**Supplementary Figure 2:** Risk of Bias in Included trials using the ROB 2.0 tool


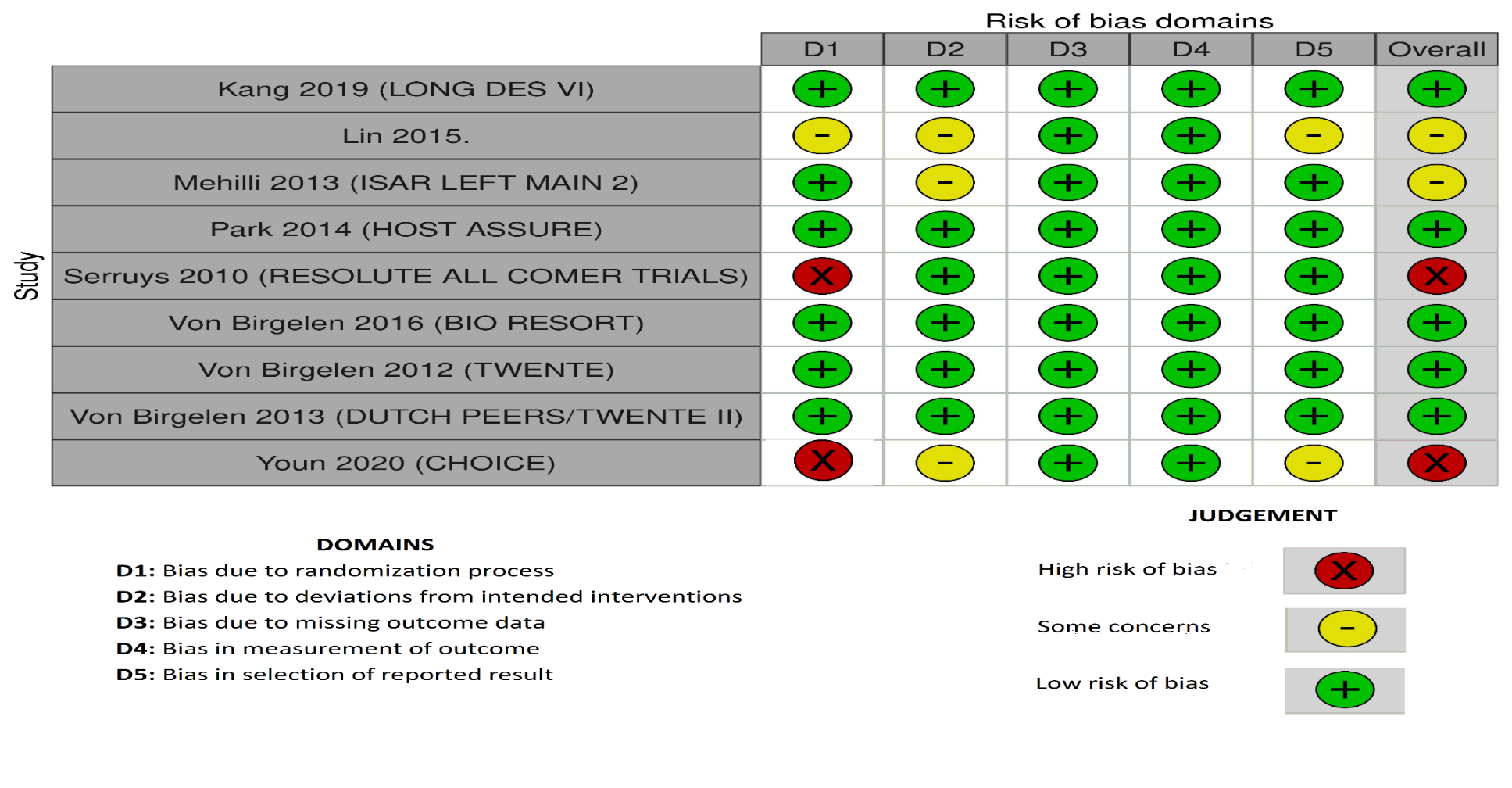


**Supplementary Figure 3:** Funnel plots for pooled outcomes at short (A=All cause death, B= cardiac death, C=MACE) intermediate (D=All cause death, E= cardiac death, F=MACE) and Long (G=All cause death, H= cardiac death, I=MACE) term follow-up


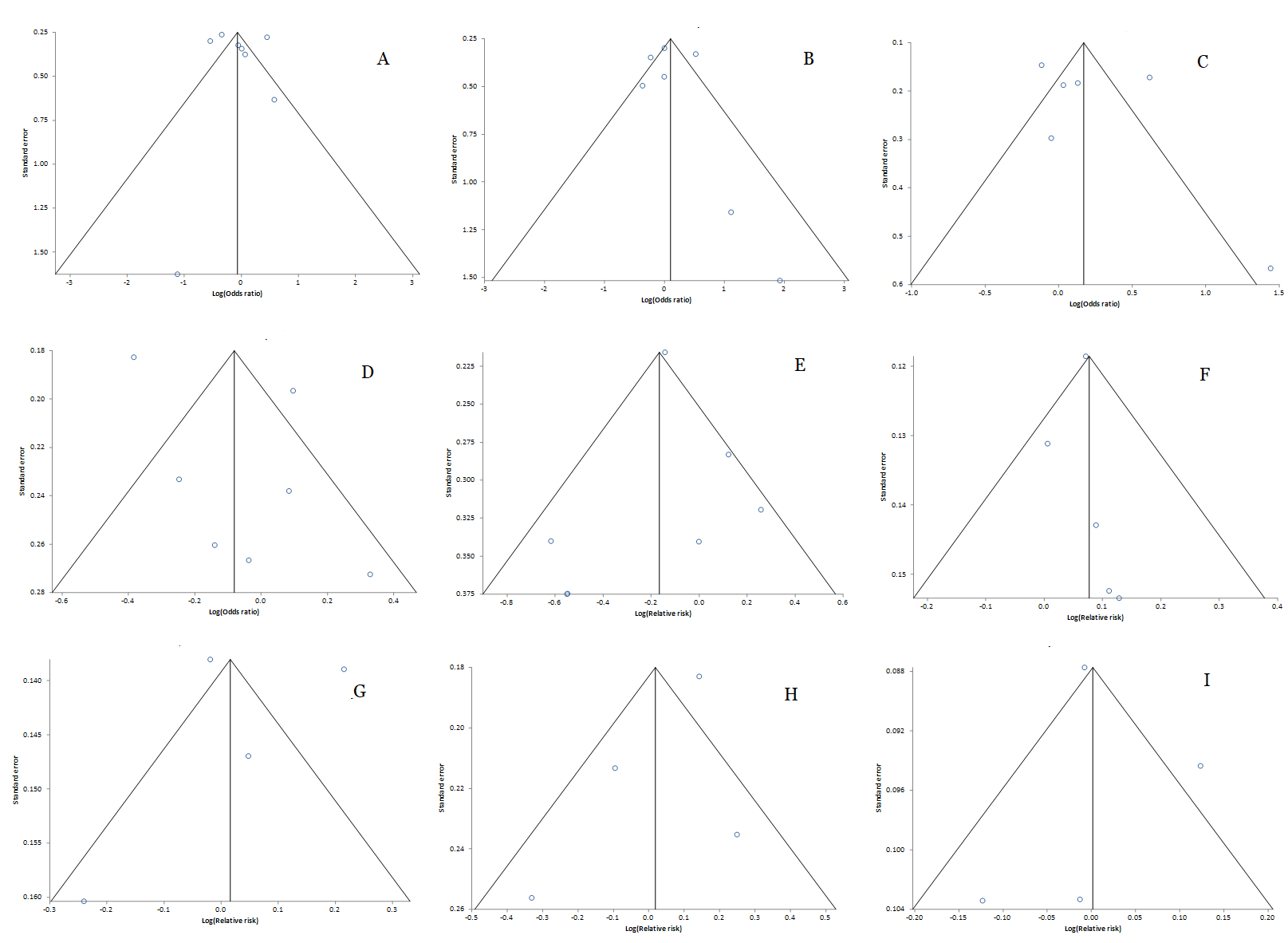


**Supplementary Figure 4**: Forest plots showing pooled results at short-term follow-up (A) Target vessel Myocardial Infraction (B) Definite or probable stent thrombosis (ST) (C) Definite stent thrombosis (ST)


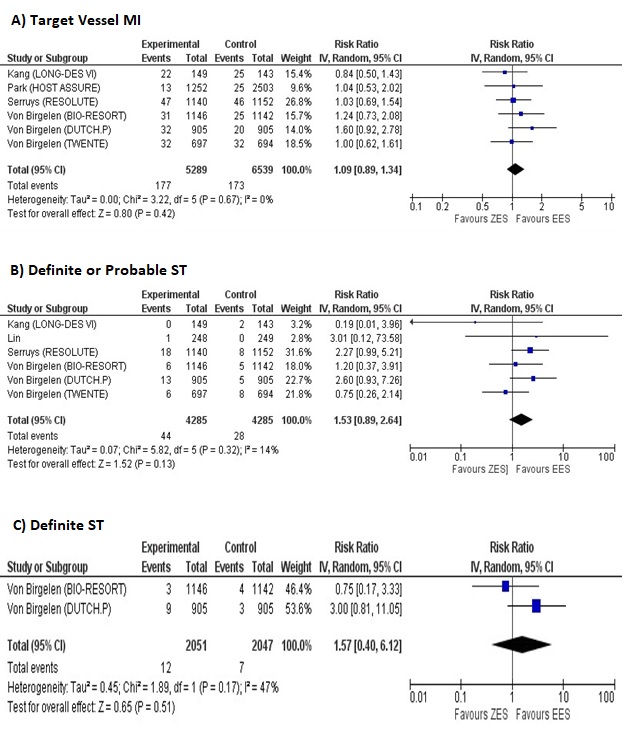


**Supplementary Figure 5**: Forest plots showing pooled results at short-term follow-up (A) Target vessel revascularization [TVR] (B) Target lesion revascularization [TLR] (C)Target vessel failure [TVF] (D) Target lesion failure [TLF]


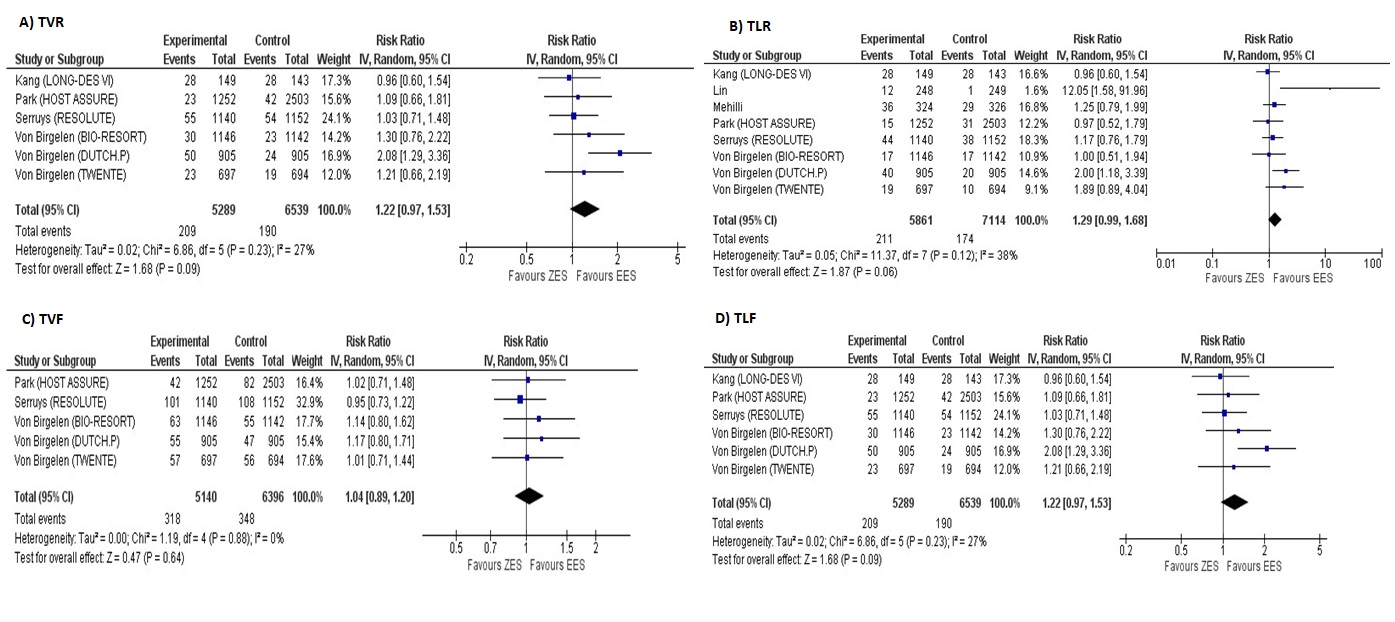


**Supplementary Figure 6:** Forest plots showing pooled results at intermediate-term follow-up (A) Target vessel Myocardial Infraction [MI] (B) Definite or probable stent thrombosis [ST] (C) Definite stent thrombosis [ST]


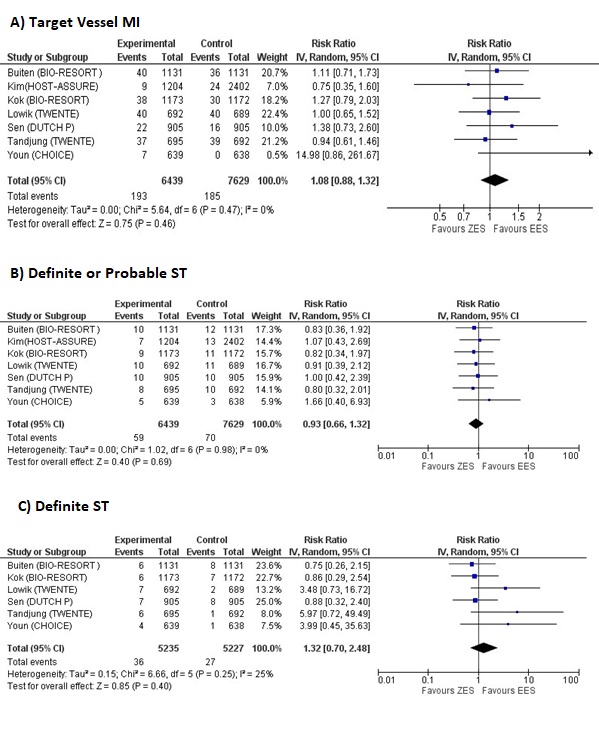


**Supplementary Figure 7:** Forest plots showing pooled results at intermediate-term follow-up (A) Target vessel revascularization [TVR] (B) Target lesion revascularization [TLR] (C)Target vessel failure [TVF] (D) Target lesion failure [TLR]


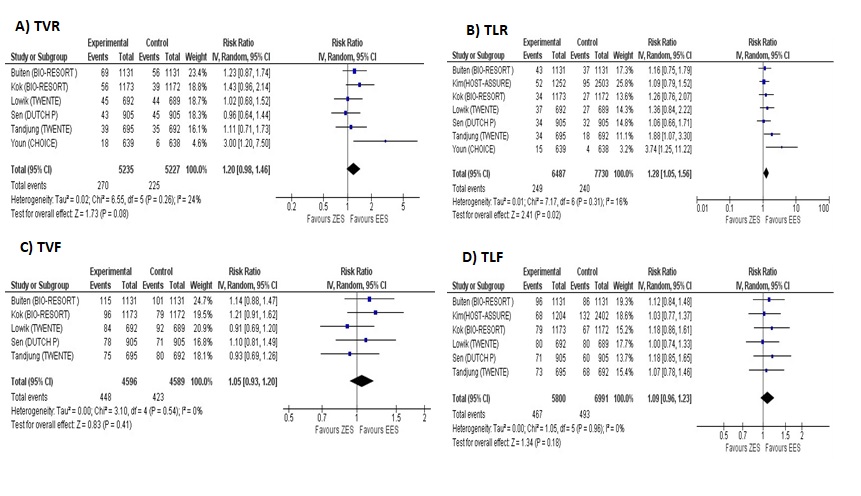


**Supplementary Figure 8:** Forest plots showing pooled results at long-term follow-up (A) Target vessel Myocardial Infraction [MI] (B) Definite or probable stent thrombosis [ST] (C) Definite stent thrombosis [ST]


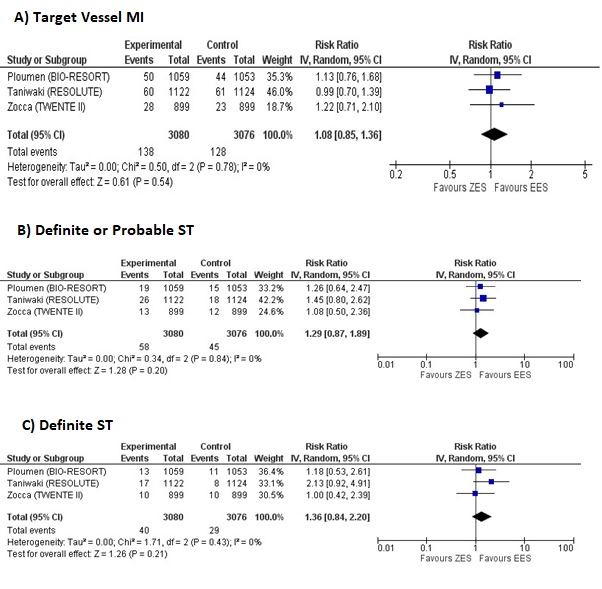


**Supplementary Figure 9:** Forest plots showing pooled results at long-term follow-up (A) Target vessel revascularization [TVR] (B) Target lesion revascularization [TLR] (C)Target vessel failure [TVF] (D) Target lesion failure [TLR]


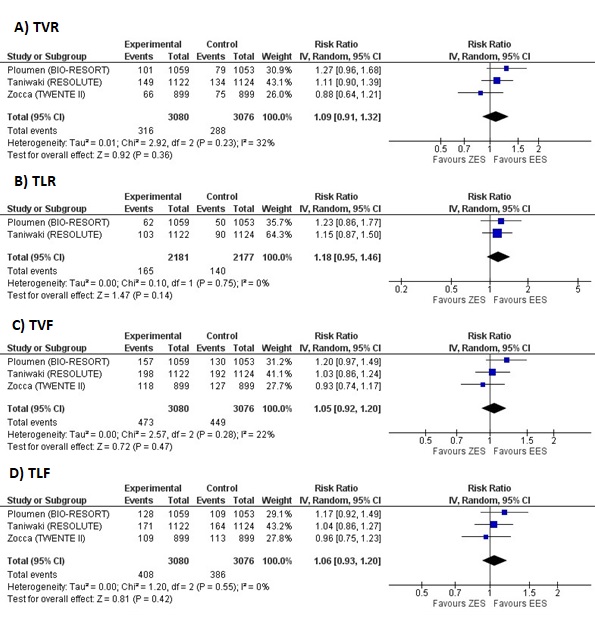


**Supplementary Figure 10:** AMSTAR 2.0 checklist


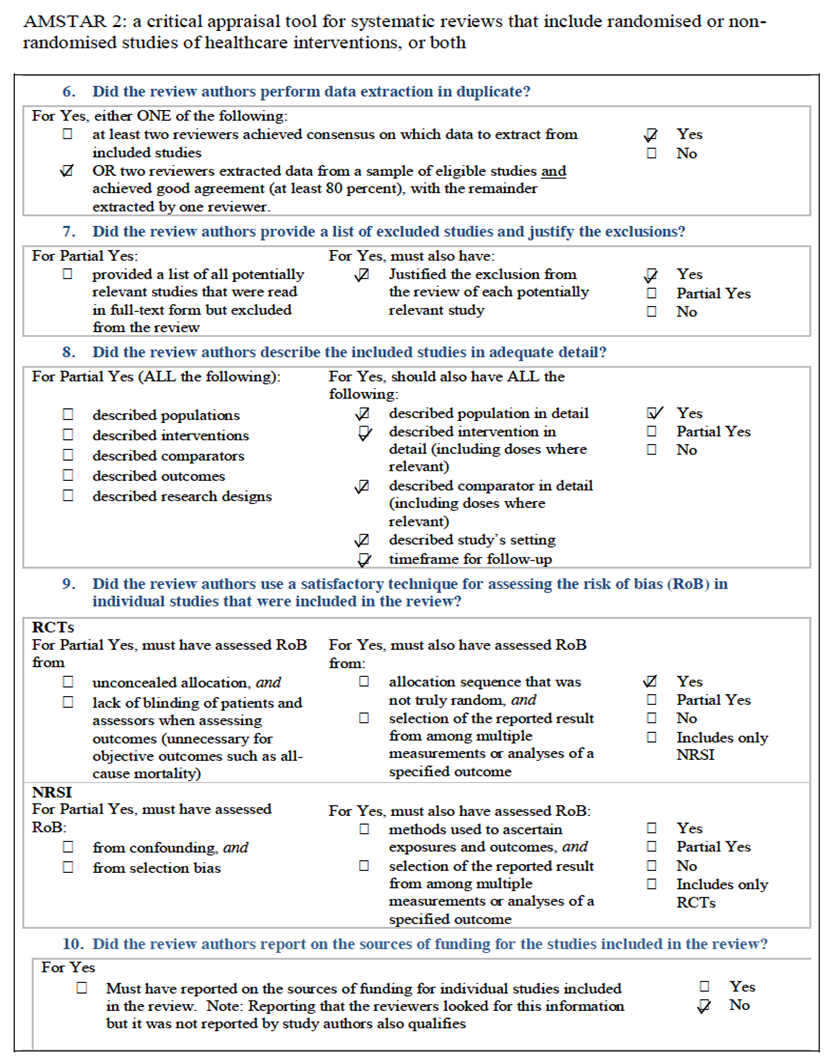


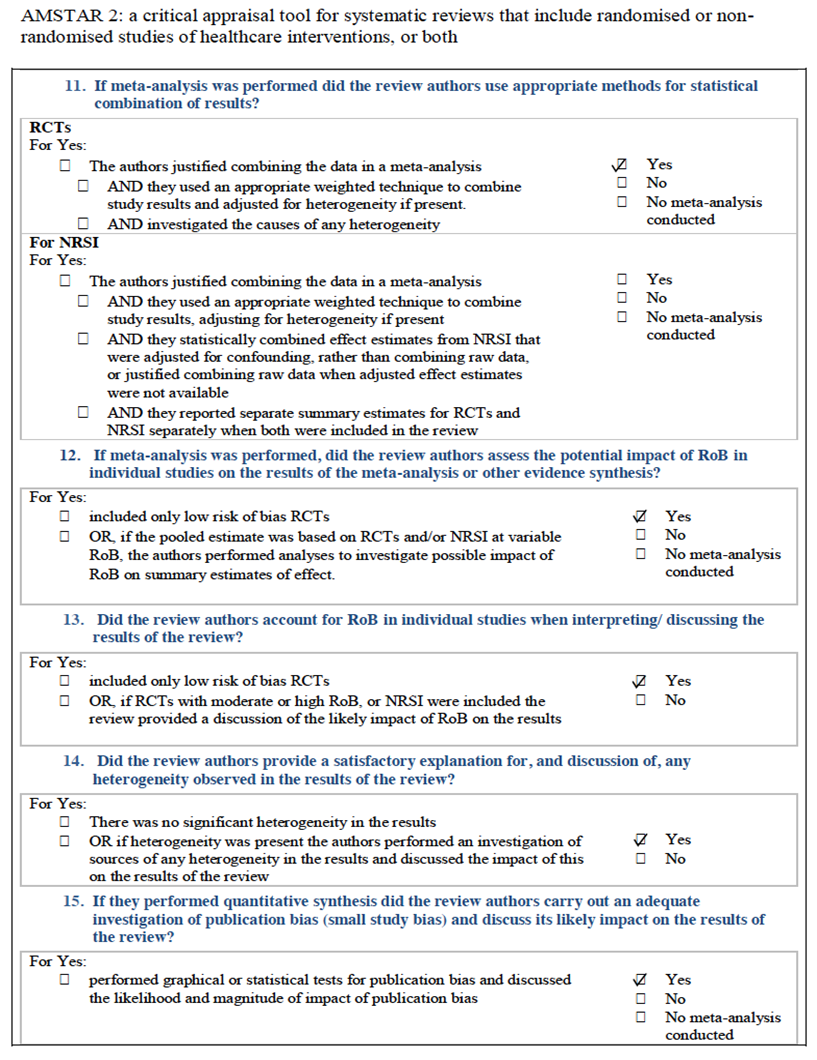


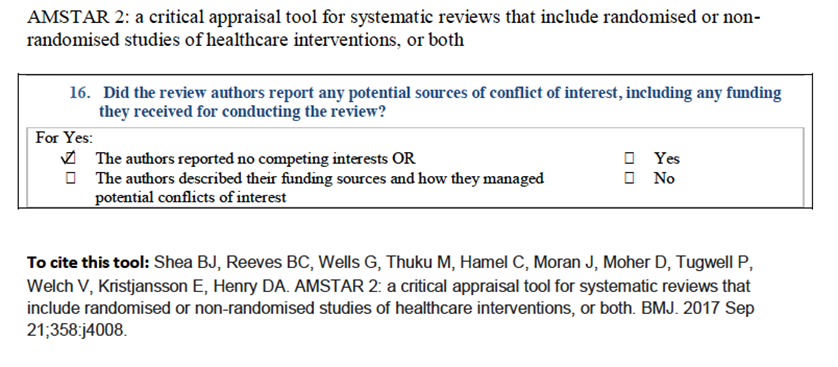


**Supplementary Table 1**: Search strategy for online databases

| Database | Search Strategy | Number of retrieved articles |
| --- | --- | --- |
| PubMed | (((((Drug eluting stents) OR (Everolimus eluting stents)) OR (Zotarolimus eluting stents)) AND (Percutaneous coronary intervention)) OR (PCI)) OR (Stents Implantation) | 509 |
| Embase | ('percutaneous coronary intervention'/exp OR 'percutaneous coronary intervention') AND ('coronary stent'/exp OR 'acs multi-link' OR 'acs multi-link duet' OR 'acs multi-link otw duet' OR 'acs multi-link otw penta' OR 'acs multi-link otw tetra' OR 'acs multi-link otw tristar' OR 'acs multi-link otw ultra' OR 'acs multi-link rx duet' OR 'acs multi-link rx penta' OR 'acs multi-link rx tetra' OR 'acs multi-link rx tristar' OR 'acs multi-link rx ultra' OR 'acs multi-link tristar' OR 'acs otw multi-link hp' OR 'acs rx multi-link' OR 'acs rx multi-link hp' OR 'ave gfx' OR 'acrobat (coronary stent)' OR 'acrobat svelte' OR 'arthos' OR 'arthos inert' OR 'arthos inert stent' OR 'avantgarde' OR 'azule' OR 'bx sonic' OR 'bx velocity' OR 'begraft coronary' OR 'blazer (stent)' OR 'chrono (coronary stent)' OR 'commander' OR 'coroflex' OR 'coroflex blue' OR 'coroflex blue neo' OR 'coroflex blue ultra' OR 'crossflex' OR 'crown (coronary stent)' OR 'driver (coronary stent)' OR 'driver (stent)' OR 'driver bms' OR 'duraflex (coronary stent)' OR 'gazelle (coronary stent)' OR 'genous (coronary stent)' OR 'jostent' OR 'lekton motion' OR 'micro stent' OR 'microdriver bms' OR 'mini vision (coronary stent)' OR 'multi-link 8 ll' OR 'multi-link 8 sv' OR 'multi-link ultra vision' OR 'multi-link zeta vision' OR 'multilink penta' OR 'multilink pixel' OR 'multilink zeta' OR 'nile croco (coronary stent)' OR 'nir (coronary stent)' OR 'palmaz-schatz' OR 'presillion' OR 'presillion plus' OR 'pro-kinetic' OR 'rebel (coronary stent)' OR 'rebel platinum' OR 's7 (coronary stent)' OR 'skylor' OR 'svelte acrobat' OR 'tecnic carbostent' OR 'titan 2' OR 'titan2' OR 'trimaxx' OR 'tryton' OR 'tsunami (coronary stent)' OR 'veriflex (coronary stent)' OR 'wiktor' OR 'xtrm-track' OR 'bestent (coronary stent)' OR 'coronary artery stent' OR 'coronary artery stents' OR 'coronary stent' OR 'coronary stents' OR 'liberte (coronary stent)' OR 'stent, coronary') AND ('everolimus eluting coronary stent'/exp OR 'promus element' OR 'promus element plus' OR 'xience alpine' OR 'xience prime' OR 'xience prime ll' OR 'xience sierra' OR 'xience skypoint' OR 'xience xpedition' OR 'xience nano' OR 'xience-v' OR 'everolimus eluting coronary stent' OR 'xience' OR 'xience v' OR 'zotarolimus eluting coronary stent'/exp OR 'endeavor (zotarolimus eluting coronary stent)' OR 'resolute integrity' OR 'resolute onyx' OR 'zomaxx' OR 'zotarolimus eluting coronary stent') AND ('randomized controlled trial'/exp OR 'controlled trial, randomized' OR 'randomised controlled study' OR 'randomised controlled trial' OR 'randomized controlled study' OR 'randomized controlled trial' OR 'trial, randomized controlled') | 845 |
| Cochrane via CENTRAL | #1 (zotarolimus eluting stents):ti,ab,kw  #2 (everolimus eluting stents):ti,ab,kw  #3 #1 OR #2  #4 MeSH descriptor: [Percutaneous Coronary Intervention] explode all trees  #5 #3 AND #4 | 543  1211  1562  8449  649 |

**Supplementary Table 2**. Detailed inclusion and exclusion criteria

| **Category** | **Inclusion criteria** | **Exclusion criteria** |
| --- | --- | --- |
| Population | - Patients undergoing stent implantation | - Non-human studies |
| Intervention/  comparators | - Zotarolimus-eluting stents vs. Everolimus-eluting stents | - Studies comparing ZES or EES with any other drug eluting stents or bare metal stents - Studies comparing ZES or EES for two different populations |
| Outcome | - Primary Outcomes: All-cause death, Cardiovascular death, and Major Adverse Cardiovascular Events - Secondary Outcomes: Target vessel myocardial infarction (MI), target vessel revascularization (TVR), target lesion revascularization (TLR), target vessel failure (TVF), target lesion failure (TLF), definite or probable stent thrombosis (ST), definite ST | - Studies not reporting any of the relevant outcomes |
| Study design | - Randomized Controlled Trials - Follow-up reports of RCTs | - Case studies, case reports, case series, Observational studies - Comments, editorials, narratives, letters to editor, opinion pieces - Systematic literature reviews and meta-analyses - Post hoc analysis of parent trials |

**Supplementary Table 3:** Definitions of pooled outcomes

| **Outcome** | **definition** |
| --- | --- |
| All cause death | Irreversible cessation of all bodily functions manifested by absence of spontaneous breathing and total loss of cerebral and cardiac functions due to any cause. |
| Cardiac death | Death due to cardiac cause |
| Major adverse cardiovascular events | A composite of non-fatal stroke, non-fatal myocardial infarction, and cardiovascular death |
| Target vessel myocardial infarction | Myocardial infarction with evidence of myocardial necrosis in the vascular territory of a previously treated target vessel. |
| Probable stent thrombosis | Any myocardial infarction with documented acute ischemia in the territory of stented segment without angiographic confirmation of stent thrombosis |
| Definite stent thrombosis | The highest level of uncertainty either angiographic or postmortem evidence of thrombotic stent occlusion |
| Target vessel revascularization | Any repeat intervention of any segment of the target vessel |
| Target lesion revascularization | Any repeat intervention for restenosed or occluded culprit target lesion |
| Target vessel failure | Composite of re-occlusion, re-stenosis and target vessel revascularization |
| Target lesion failure | Composite of target lesion revascularization, myocardial infarction or cardiac death related to target vessel. |

**Supplementary Table 4:** Reason for exclusion for studies during full length screening

| Author Name/ Year | Study Title | Reason of exclusion |
| --- | --- | --- |
| Kandzari 2011 | Late-term clinical outcomes with zotarolimus- and sirolimus-eluting stents. 5-year follow-up of the ENDEAVOR III (A Randomized Controlled Trial of the Medtronic Endeavor Drug [ABT-578] Eluting Coronary Stent System Versus the Cypher Sirolimus-Eluting Coronary Stent System in De Novo Native Coronary Artery Lesions) | Comparison of ZES and sirolimus-eluting-stents (SES) |
| Kirtane 2013 | The “final” 5-year follow-up from the ENDEAVOR IV trial comparing a zotarolimus-eluting stent with a paclitaxel-eluting stent | Comparison of ZES and PES |
| Byrne 2010 | 2-year clinical and angiographic outcomes from a randomized trial of polymer-free dual drug-eluting stents versus polymer-based Cypher and Endeavor [corrected] drug-eluting stents | Compared ZES with dual drug eluting stents (DES) and SES |
| Valgimigli 2014 | Two-year outcomes after first- or second-generation drug-eluting or bare-metal stent implantation in all-comer patients undergoing percutaneous coronary intervention: a pre-specified analysis from the PRODIGY study (PRO longing Dual Antiplatelet Treatment After Grading stent-induced Intimal hyperplasia study | Data for assessed clinical outcomes not reported |
| Maeng 2014 | Differential clinical outcomes after 1 year versus 5 years in a randomized comparison of zotarolimus-eluting and sirolimus-eluting coronary stents (the SORT OUT III study): a multicenter, open-label, randomized superiority trial | Comparison of ZES with SES |
| Jang 2013 | Differential long-term outcomes of zotarolimus-eluting stents compared with sirolimus-eluting and paclitaxel-eluting stents in diabetic and nondiabetic patients: two-year subgroup analysis of the ZEST randomized trial | Comparison of ZES with SES and paclitaxel eluting stents (PES). |
| Teeuwen 2015 | Tree-year clinical outcome in the Primary Stenting of Totally Occluded Native Coronary Arteries III (PRISON III) trial: a randomized comparison between sirolimus-eluting stent implantation and zotarolimus-eluting stent implantation for the  treatment of total coronary occlusions | Comparison of ZES and SES |
| Chevalier 2013 | A randomized, controlled, multicenter trial to evaluate the safety and efficacy of Zotarolimus- vs. Paclitaxel-eluting stents in de novo occlusive lesions in coronary arteries: five-year results from ZOMAXX I trial | Comparison of ZES and PES |
| Fahrni 2020 | Long-Term Results After Drug-Eluting Versus Bare-Metal Stent Implantation in Saphenous Vein Grafts: Randomized Controlled Trial | Comparison of DES and Bare-metal stents |
| Winter 2018 | A sirolimus-eluting bioabsorbable polymer-coated stent (MiStent) versus an everolimus-eluting durable polymer stent (Xience) after percutaneous coronary intervention (DESSOLVE III): a randomized, single-blind, multicentre, non-inferiority, phase 3 trial | Comparison of SES and EES |
| Tousek 2022 | Comparison of a Bioresorbable, Magnesium-Based Sirolimus-Eluting Stent with a Permanent, Everolimus-Eluting Metallic Stent for Treating Patients with Acute Coronary Syndrome: the PRAGUE-22 Study | Comparison of SES and EES |
| Buiten 2020 | Thin Composite-Wire-Strut Zotarolimus-Eluting Stents Versus Ultrathin-Strut Sirolimus-Eluting Stents in BIONYX at 2 Years | Comparison of SES and ZES |
| Zaman 2019 | Safety and efficacy of a sirolimus-eluting coronary stent with ultra-thin strut for treatment of atherosclerotic lesions (TALENT): a prospective multicenter randomized controlled trial | Comparison of SES and ZES |
| Hansen 2022 | Impact of diabetes on 1-year clinical outcome in patients undergoing revascularization with the BioFreedom stents or the Orsiro stents from the SORT OUT IX trial | Not fulfill PICO criteria |
| Hong 2021 | 1-Month Dual-Antiplatelet Therapy Followed by Aspirin Monotherapy After Polymer-Free Drug-Coated Stent Implantation: One-Month DAPT Trial | No data regarding ZES and EES |
| Winter 2022 | Sirolimus-eluting stents with ultrathin struts versus everolimus-eluting stents for patients undergoing percutaneous coronary intervention: final three-year results of the TALENT trial | Comparison of SES with ZES |
| Wijnbergen 2014 | Long-term comparison of sirolimus-eluting and bare-metal stents in ST-segment elevation myocardial infarction | Comparison of SES and bare-metal stents |
| Garcia 2015 | Comparison between diabetic and non-diabetic patients after successful percutaneous coronary intervention for chronic total occlusions in the drug-eluting stent era | No data reported for ZES and EES |
| Iglesias 2022 | Five-Year Outcomes with Biodegradable-Polymer Sirolimus-Eluting Stents Versus Durable-Polymer Everolimus-Eluting Stents in Patients with Acute Coronary Syndrome: A Subgroup Analysis of the BIOSCIENCE Trial | Comparison of SES and EES |
| Pedersen 2014 | Drug-eluting stents and bare metal stents in patients with NSTE-ACS: 2-year outcome from the randomised BASKET-PROVE trial | Clinical outcomes with ZES and EES not assessed |
| Yoon 2021 | BioMatrix versus Orsiro biodegradable polymer stents in all-comer patients with coronary artery disease: the multicentre, randomised BIODEGRADE trial | Comparison of two biodegradable polymer stents |
| Eriksen 2023 | Everolimus-eluting bioresorbable scaffold versus everolimus-eluting metallic stent in primary percutaneous coronary intervention of ST-segment elevation myocardial infarction: a randomized controlled trial | Comparison of two different kinds of EES |
| Rozemeijer 2019 | Randomized All-Comers Evaluation of a Permanent Polymer Zotarolimus-Eluting Stent Versus a Polymer-Free Amphilimus-Eluting Stent | Comparison of ZES and amphilimus eluting stents |
| Von Birgelem 2016 | Five year outcomes after implantation of zotarolimus eluting vs everolimus eluting stents in randomized trial participants and non-enrolled eligible patients: A secondary analysis of an RCT | Secondary analysis of TWENTE |

**Supplementary table 5:** Details of included trials and their follow-up studies

| **Trial/Study name** | **Follow-up studies** |
| --- | --- |
| Park 2014 (HOST ASSURE) | Kim 2019 (3-year follow up) |
| Mehili 2013 (ISAR-LEFT-MAIN 2) | None |
| Von Birglen 2013 (DUTCH PEERS/ TWENTE II) | Sen 2015 (2-year follow-up)  Zocca 2018 (5-year follow-up) |
| Von Birglen 2012 (TWENTE) | Tanjdung 2013 (2-year follow-up)  Lowik 2014 (3-year follow-up) |
| Von Birglen 2016 (BIO-RESORT) | Kok 2018 (2-year follow-up)  Buiten 2019 (3-year follow-up)  Ploumen 2022 (5-year follow-up) |
| Serruys 2010 (Resolute All Comers trial) | Taniwaki 2014 ( 4 year follow-up) |
| Youn 2020 (Choice) | None |
| Kang 2019 (LONG-DES VI) | None |
| Lin 2015 | None |

**Supplementary Table 6:** Publication bias using Eggers and Begg’s test for short term follow-up

| OUTCOMES | Egger’s test | Begg’s test | PUBLICATION BIAS |
| --- | --- | --- | --- |
| Target vessel failure | 0.10 | 0.48 | No significant publication bias |
| Target lesion failure | 0.79 | 0.71 | No significant publication bias |
| Death due to any cause | 0.93 | 0.54 | No significant publication bias |
| Cardiac death | 0.29 | 0.56 | No significant publication bias |
| Target vessel MI | 0.96 | 0.99 | No significant publication bias |
| Target vessel revascularization | 0.99 | 0.81 | No significant publication bias |
| Target lesion revascularization | 0.12 | 0.39 | No significant publication bias |
| MACE | 0.36 | 0.71 | No significant publication bias |
| Definite or probable ST | 0.43 | 0.46 | No significant publication bias |
| Definite ST | Not assessable^*^ | Not assessable^*^ | Not assessable^*^ |

^*^Cannot be assessed due to limited number of studies reporting the particular outcome

**Supplementary Table 7:** Publication bias using Eggers and Begg’s test for long term follow-up

| OUTCOMES | Egger’s test | Begg’s test | PUBLICATION BIAS |
| --- | --- | --- | --- |
| Target vessel failure | 0.75 | 0.81 | No significant publication bias |
| Target lesion failure | 0.42 | 0.27 | No significant publication bias |
| Death due to any cause | 0.24 | 0.23 | No significant publication bias |
| Cardiac death | 0.38 | 0.23 | No significant publication bias |
| Target vessel MI | 0.13 | 0.38 | No significant publication bias |
| Target vessel revascularization | 0.13 | 0.99 | No significant publication bias |
| Target lesion revascularization | 0.01 | 0.01 | Significant publication bias |
| MACE | 0.26 | 0.08 | No significant publication bias |
| Definite or probable ST | 0.02 | 0.56 | No significant publication bias |
| Definite ST | 0.01 | 0.13 | No significant publication bias |

**Supplementary Table 8:** Publication bias using Eggers and Begg’s test for long term follow-up

| OUTCOMES | Egger’s TEST | Begg’s test | PUBLICATION BIAS |
| --- | --- | --- | --- |
| Target vessel failure | 0.61 | 0.08 | No significant publication bias |
| Target lesion failure | 0.77 | 0.08 | No significant publication bias |
| Death due to any cause | 0.21 | 0.33 | No significant publication bias |
| Cardiac death | 0.47 | 0.33 | No significant publication bias |
| Target vessel MI | 0.12 | 0.33 | No significant publication bias |
| Target vessel revascularization | 0.39 | 0.33 | No significant publication bias |
| Target lesion revascularization | Not assessable^*^ | Not assessable^*^ | Not assessable^*^ |
| MACE | 0.55 | 0.08 | No significant publication bias |
| Definite or probable ST | 0.09 | 0.08 | No significant publication bias |
| Definite ST | 0.75 | 0.73 | No significant publication bias |

^*^Cannot be assessed due to limited number of studies reporting the particular outcome
